# When speaking of the experience, do not leave out the experiencer: on self and magnitude

**DOI:** 10.3389/fpsyg.2013.00303

**Published:** 2013-05-28

**Authors:** Shahar Arzy

**Affiliations:** ^1^Department of Neurology, Hadassah Hebrew University Medical CenterJerusalem, Israel; ^2^Faculty of Medicine, Hebrew UniversityJerusalem, Israel

Memory, motor-control, attention, learning, navigation, emotion, and perception are among the foundations of cognitive neurosciences. For many years, these have been studied separately, as distinct functions (Fodor, 2000). Recently, several veins of research have lead to the idea that different cognitive faculties may be handled by similar neurocognitive mechanisms. Likewise, Buzsáki and Moser proposed that a range of interacting cell types (such as “place cells,” “grid cells” or “time cells”), which support navigation, may also play a role in memory (Buzsáki and Moser, 2013). Moreover, these prominent researchers have suggested that navigation and memory rely on two fundamental mechanisms: one that is more allocentric, related to representations of landmarks in the environment, and another that is egocentric, self-referenced (Buzsáki and Moser, 2013). Similarly to navigation, memory encompasses autobiographical memory, related to events that happened to the experiencer (self-referenced), and semantic memory of events that the experiencer “knows”. Perception may be taken from a self-referenced first-person-perspective or from a third-person-perspective. Correspondingly, in the affective plane, emotion may be self-referenced, reflecting the experiencer own-feelings, or may be dominated by a third-person-perspective, when the experiencer is absorbed in the life of others (Zinck, 2008).

Another vein of research, which pointed to cross-modalities, relates to “mental-lines.” Experiments on mental number scaling in archaic cultures or children have revealed that humans represent numbers along a logarithmic scale, termed “mental-number-line” (Dehaene and Cohen, 1995; Dehaene et al., 1999, 2008). Human experience numbers according to the resolution of perception: the perceived resolution decreases as numbers increase, yielding logarithmic scale. Logarithmic distribution was shown to fit the relation between temporal-distance of the experiencer from the experience and memory retention (Rubin and Schulkind, 1997; Spreng and Levine, 2006). Moreover, cognitive performance was found to decrease logarithmically as temporal-distance to the event increased (Arzy et al., 2009a). Emotional expression was also found to be represented by a mental-magnitude-line (Holmes and Lourenco, 2011). It is proposed that these common patterns of magnitudes are related to the self-referenced (spatial) processing of the different domains.

The temporo-parietal junction (TPJ) is believed to play a special role in these self-referenced magnitude-related processing. The TPJ was found to be implicated in processing the mental-number-line (Göbel et al., 2001) with respect to quantity, numbers, or spatial attention (Dehaene et al., 2003), and likewise may be involved in other mental-magnitude-lines. However, the TPJ is known to be involved in many self-referenced functions including agency, ownership, perspective-taking and autobiographical memory which are not necessarily related to magnitude (Blanke and Arzy, 2005). Likewise, in a couple of investigation of the mental-time-line (Arzy et al., 2009b), activation at the right TPJ showed a symmetrical distribution of brain activity as a function of the temporal-distance of events from the present time: activation was increased for closer events than for more distant events (both in past and future). The TPJ was also found to play a special role in coordinating the relation between one's self-location in space and different external reference points (Ruby and Decety, 2001; Vogeley and Fink, 2003). In the personal/social domain, the TPJ was found to coordinate the relation between mentalizing oneself and others (Lombardo et al., 2010).

Taken together, this suggests that different aspects of the subjective experience should be regarded in relation to the experiencing self. Self-related mentalization may have a specific logarithmic pattern, reflected as a “mental-line.” The temporo-parietal junction may play a special role in mediating these self-referenced functions in the different domains.
